# Supplementary material for: Comprehensive whole-genome sequencing reveals origins of mutational signatures associated with aging, mismatch repair deficiency and temozolomide chemotherapy
Source: Nucleic Acids Res. 2024 Dec 5;53(1):gkae1122. doi: 10.1093/nar/gkae1122 (PMC11724276; doi:10.1093/nar/gkae1122)
Supplement: gkae1122_Supplemental_Files [file gkae1122_supplemental_files.zip › Supplementary Figures.pdf]

Supplementary Figure 1

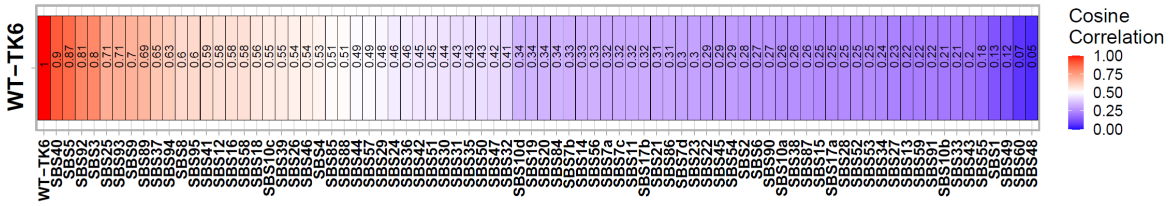

Supplementary Figure S1. Cosine correlation of the TK6 WT spontaneous mutational spectrum to all COSMIC mutational signatures.

Supplementary Figure 2

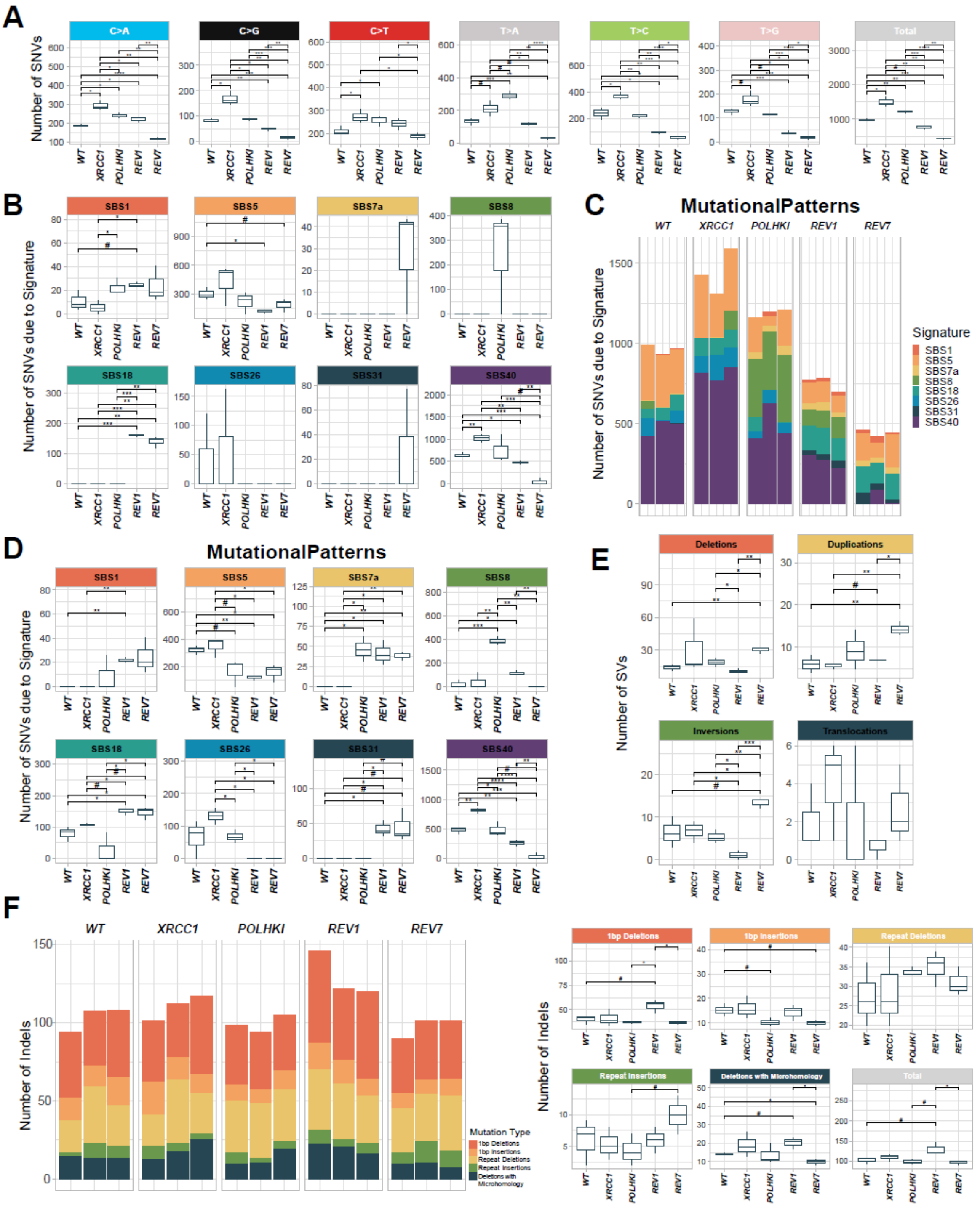

Supplementary Figure S2. Box plots of spontaneous mutations. Box plots indicate the statistical significance of differences in mutation numbers among the cell lines in Figure 2. Panels **A**, **B**, and **E** correspond to panels **A**, **C**, and **D** of Figure 2, respectively. **(C)** and **(D)** Related to Figure 2C. Mutational profiles were decomposed into SBS signatures using the alternative MutationalPatterns tool instead of SigProfilerExtractor, which was used in Figure 2C. **(E)** Numbers of SV accumulated in different cell lines after ~180 cell doublings (10 passages). Significant differences are indicated by # and * (# p value ≤0.1, * p value ≤ 0.05, ** p value ≤ 0.01, *** p value ≤ 0.001, t-test). **(F)** Numbers of indels accumulated in different

cell lines after ~180 cell doublings (10 passages). Significant differences are indicated by # and * (# p value $\leq$0.1, * p value $\leq$ 0.05, ** p value $\leq$ 0.01, *** p value $\leq$ 0.001, t-test).

Supplementary Figure 3

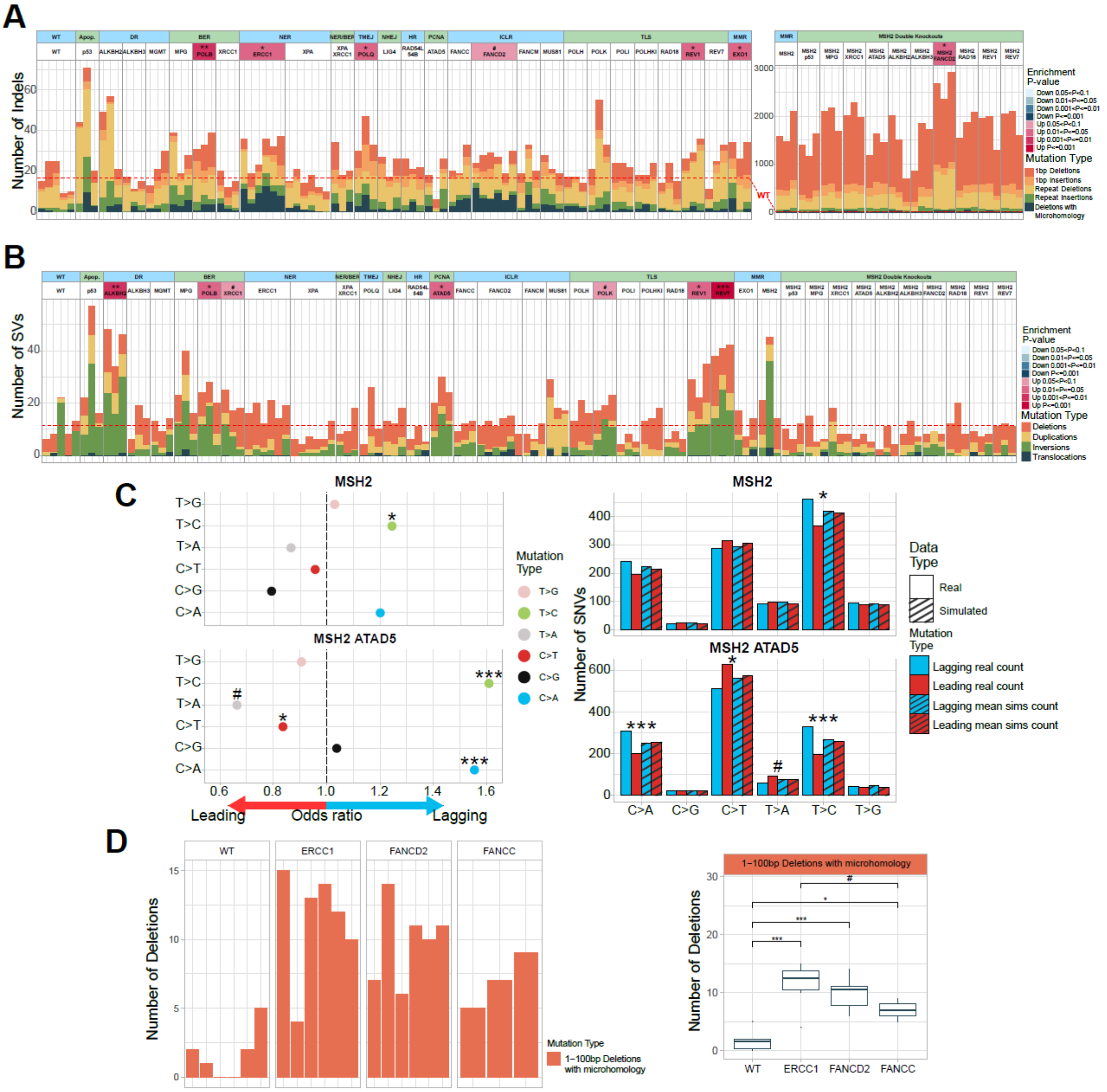

Supplementary Figure S3. Spontaneous mutagenesis in the collection of TK6 DNA repair mutants. Numbers of indels **(A)** and SVs **(B)** accumulated after 18 cell doublings in individual subclones. The dashed line corresponds to an average of WT. The statistical tests on the left panel are compared to the WT lines, and the right panel is compared to the *MSH2-/-* lines. **(C)** Replication strand bias in *MSH2-/-* and *MSH2-/- ATAD5-/-* lines. **(D)** Deletions with microhomology in *ERCC1-/-*, *FANCD2-/-*, and *FANCC-/-* lines. Numbers of 1-100 bp deletions flanked by microhomologies. Significant differences are indicated by # and * (# p value ≤0.1, * p value ≤ 0.05, ** p value ≤ 0.01, *** p value ≤ 0.001, t-test).

Supplementary Figure 4

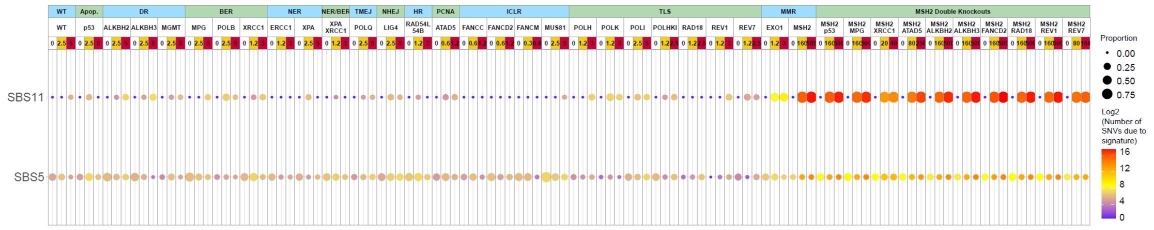

Supplementary Figure S4. Signatures SBS5 and SBS11 induced by TMZ. The prominence of signatures SBS5 and SBS11 in the TMZ-induced mutational profiles. The size of the dot represents the proportion of SNVs, and the color represents the log2 of the number of SNVs due to signature SBS5 or SBS11.

Supplementary Figure 5

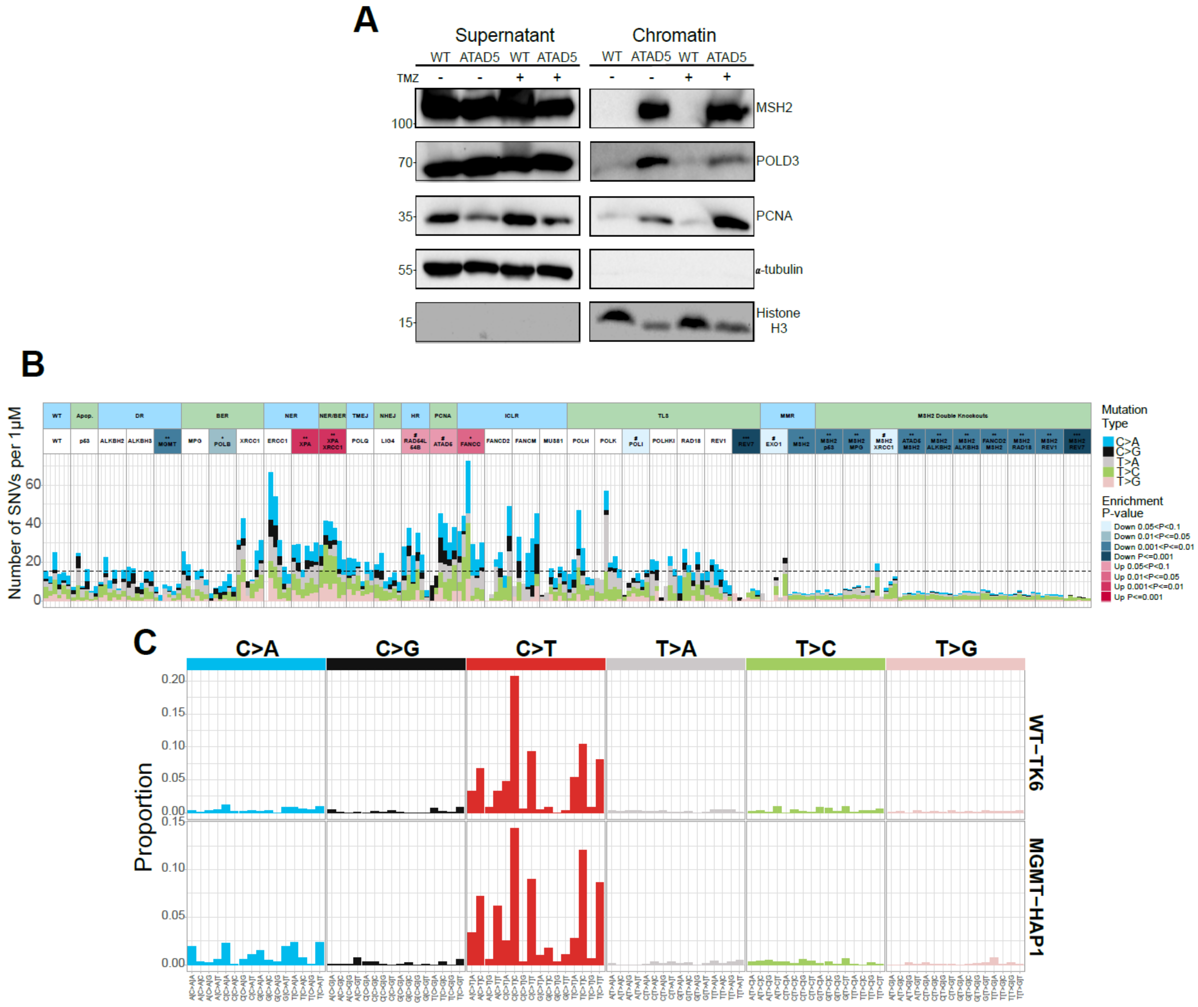

Supplementary Figure S5. TMZ-induced mutations in the collection of TK6 DNA repair mutants. **(A)** Chromatin fractionation assay. Cells were treated with LD_{90} concentration of TMZ (1.25 microM for *ATAD5-/-*, 5 microM for WT) for 24 hours. **(B)** Numbers of non C>T substitutions induced per microM TMZ. **(C)** Comparison of the TMZ-induced mutational spectra in WT TK6 and *MGMT-* HAP1 lines.

Supplementary Figure 6

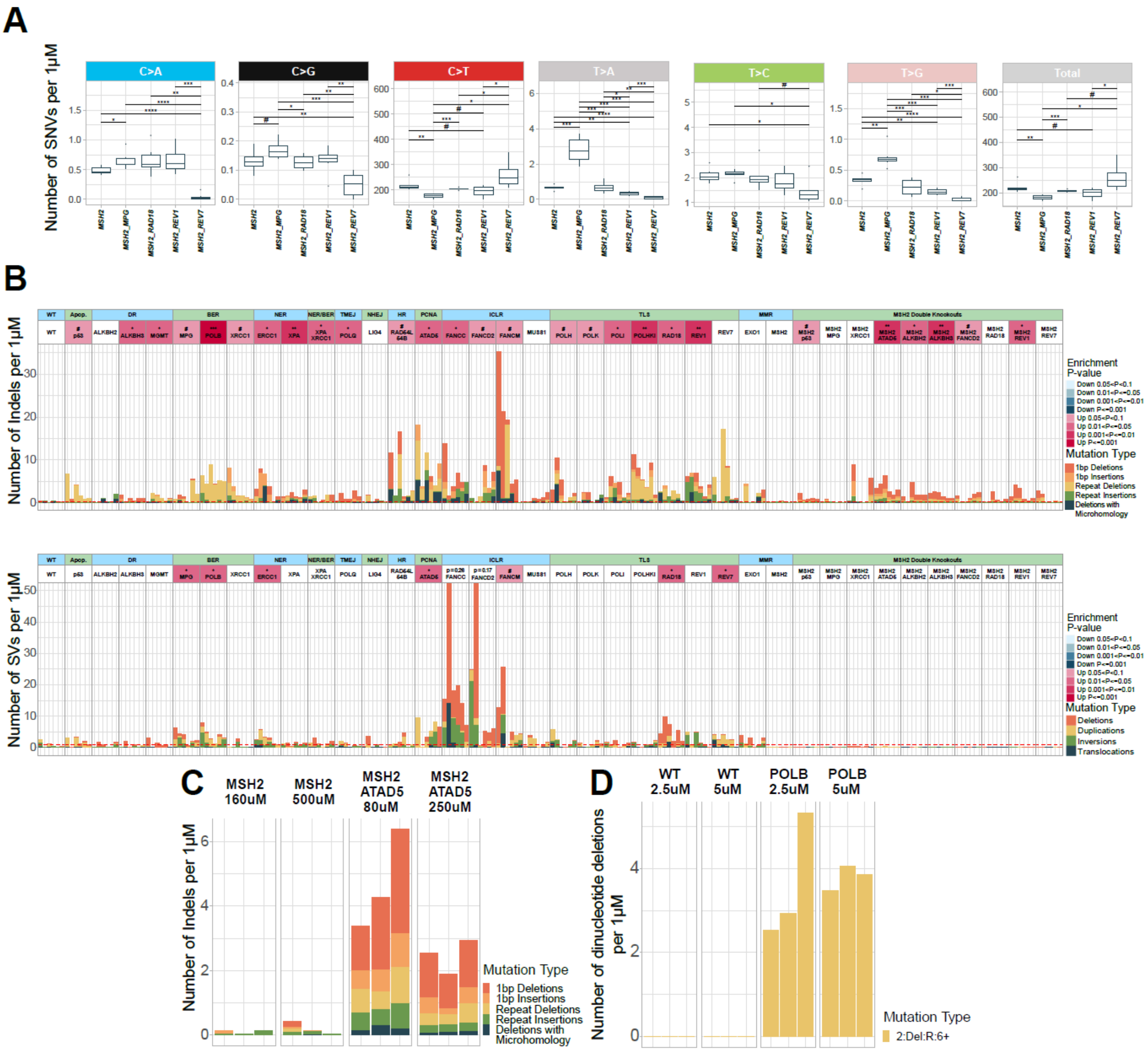

Supplementary Figure S6. TMZ-induced mutations in the collection of TK6 DNA repair mutants. **(A)** Box plots indicate the statistical significance of differences in mutation numbers among the cell lines in Figure 7C. Significant differences are indicated by # and * (# p value ≤0.1, * p value ≤ 0.05, ** p value ≤ 0.01, *** p value ≤ 0.001, t-test). **(B)** Numbers of indels (upper panel) and SVs (lower panel) induced per microM TMZ after subtracting untreated background. The dashed line corresponds to an average of WT. The statistical tests are in comparison to the WT line. **(C)** Numbers of indels induced per microM TMZ after subtraction of untreated background in *MSH2-/- ATAD5-/-* line. **(D)** Numbers of dinucleotide deletions in poly A repeats longer than 12 nt induced in the POLB-/- line per microM TMZ after subtraction of untreated background.

Supplementary Figure 7

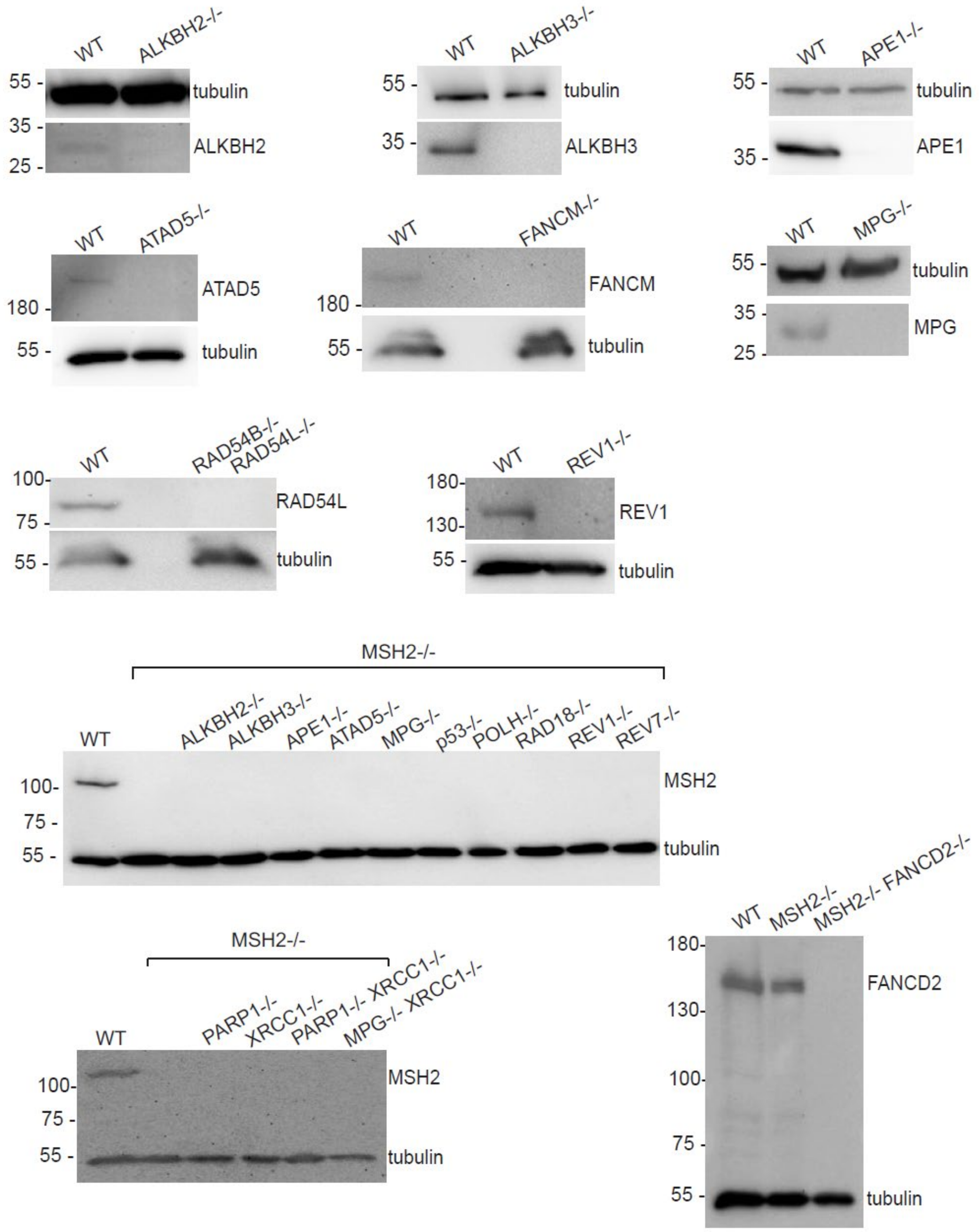

Supplementary Figure S7. Western blots demonstrating the absence of the targeted proteins in the knockout cell lines generated in this study.

Supplementary Figure 8

A

B

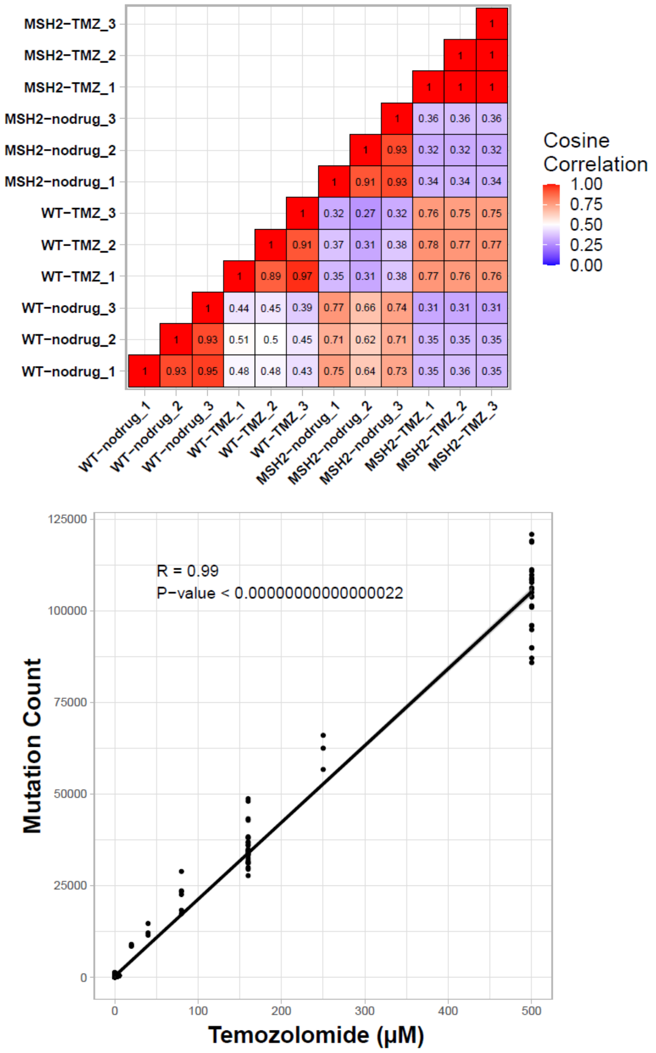

Supplementary Figure S8. Consistency of mutational spectra across replicates. (A) Cosine correlations among the SNV patterns of three replicates obtained with the same cell line and condition. (B) A linear correlation between TMZ concentration and mutational burden compiling the data for all the cell lines.

Supplementary Figure 9

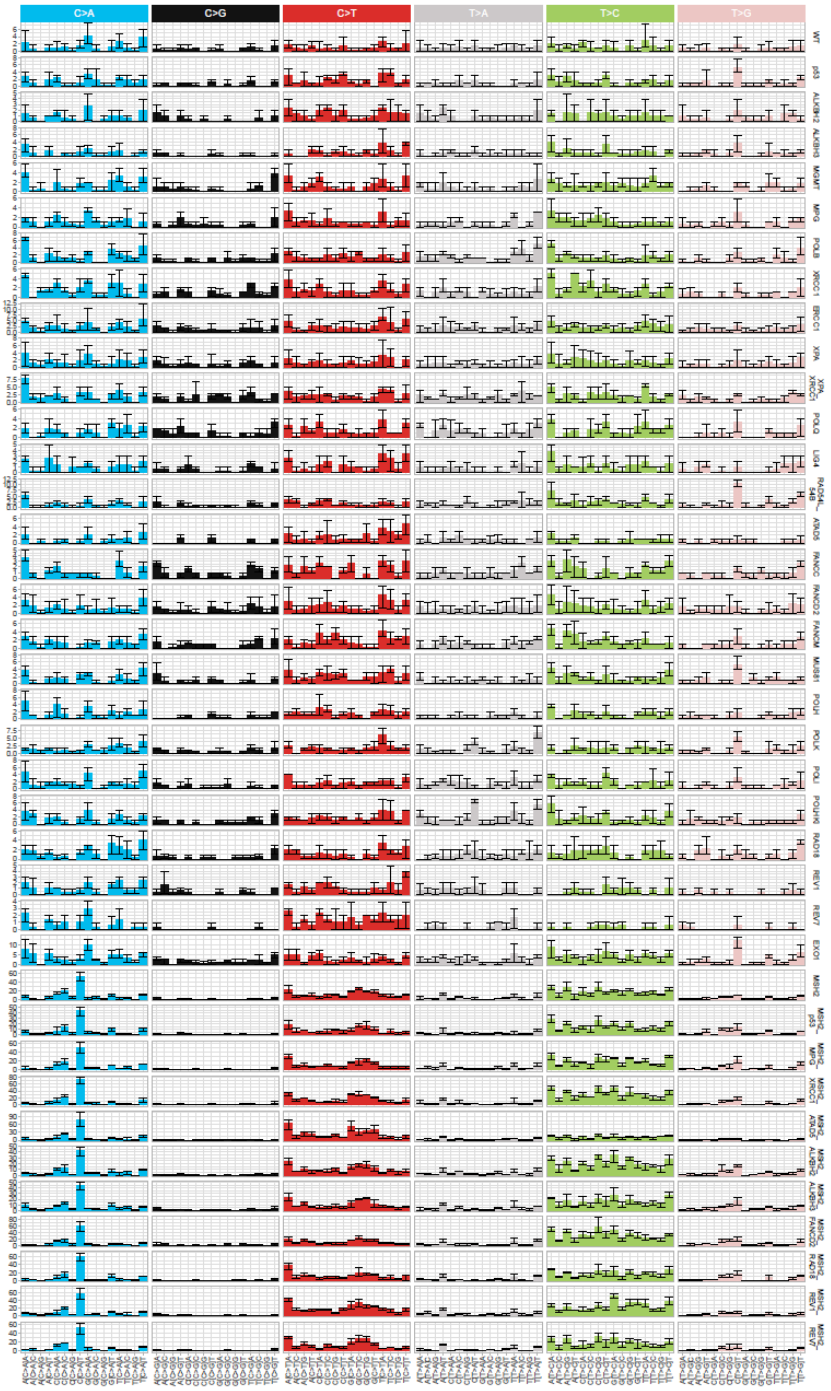

Supplementary Figure S9. Spontaneous SNV patterns of all the cell lines used in this study with error bars demonstrating variability among the replicates.
