# Supplementary material for: Comprehensive whole-genome sequencing reveals origins of mutational signatures associated with aging, mismatch repair deficiency and temozolomide chemotherapy
Source: Nucleic Acids Res. 2024 Dec 5;53(1):gkae1122. doi: 10.1093/nar/gkae1122 (PMC11724276; doi:10.1093/nar/gkae1122)

Figure 6C

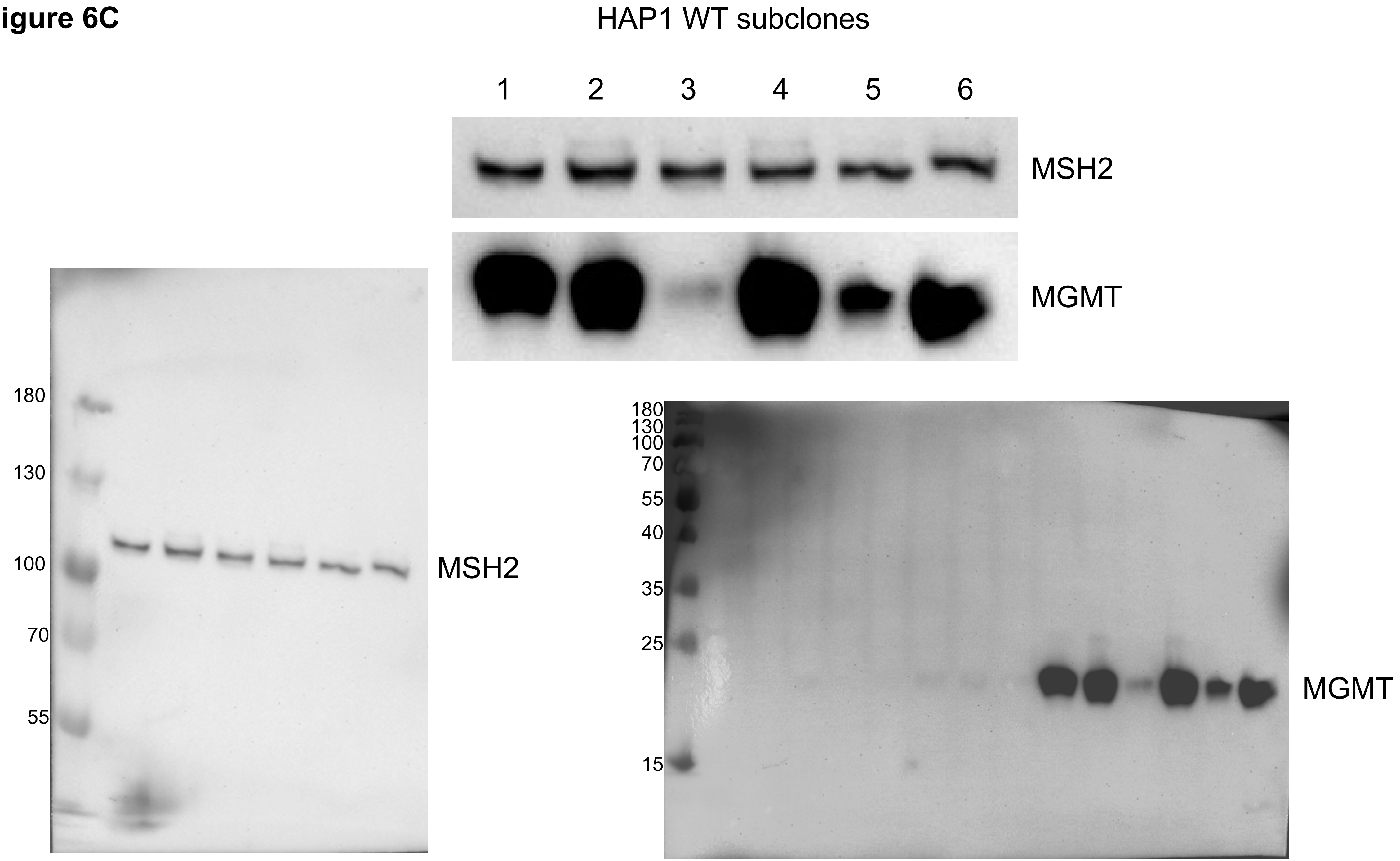
HAP1 WT subclones
1
2
3
4
5
6
MSH2
MGMT
180
130
100
70
55
MSH2
180
130
100
70
55
40
35
25
15
MGMT

Supplementary Figure 5A

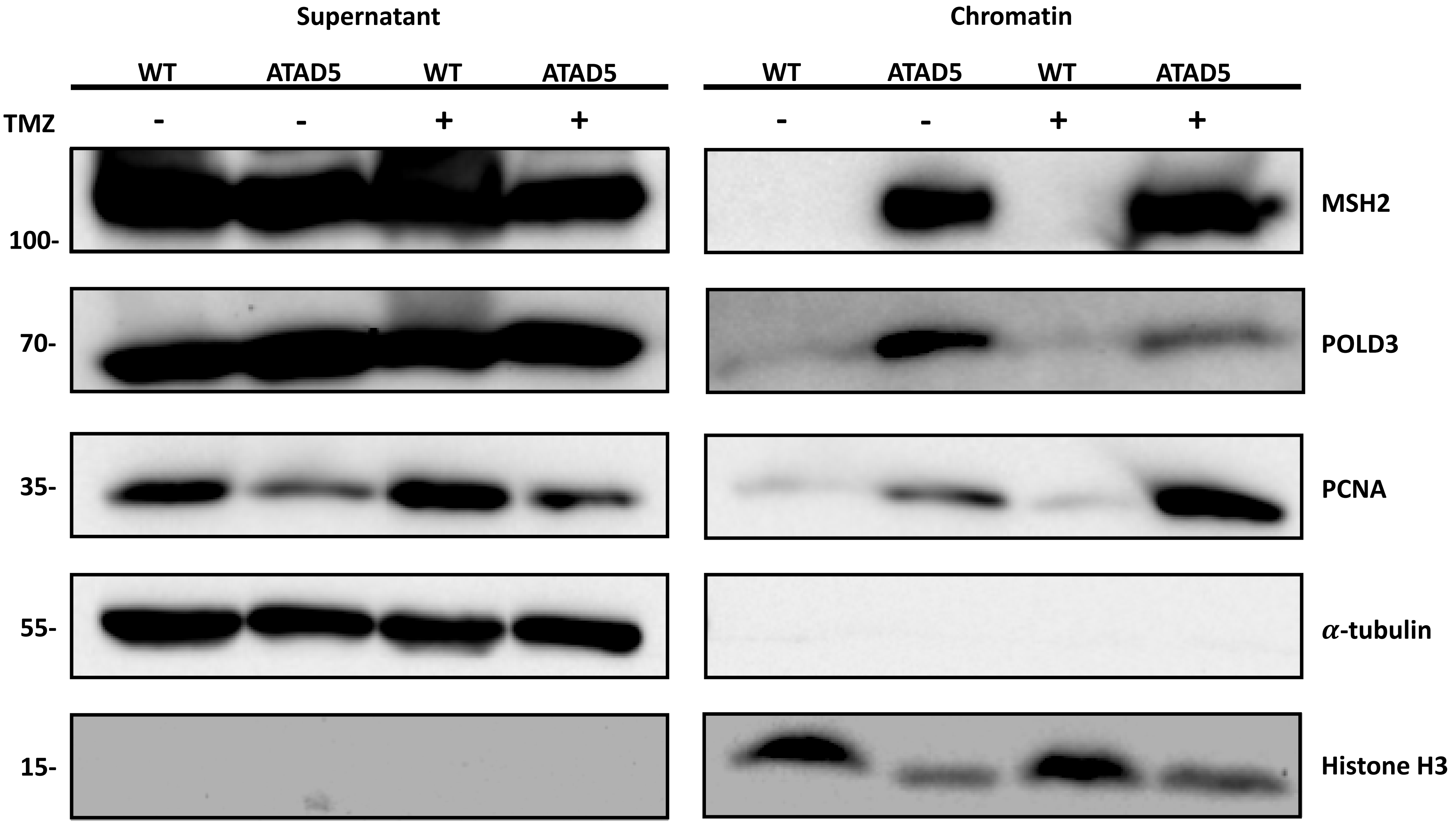

Supplementary Figure 5A

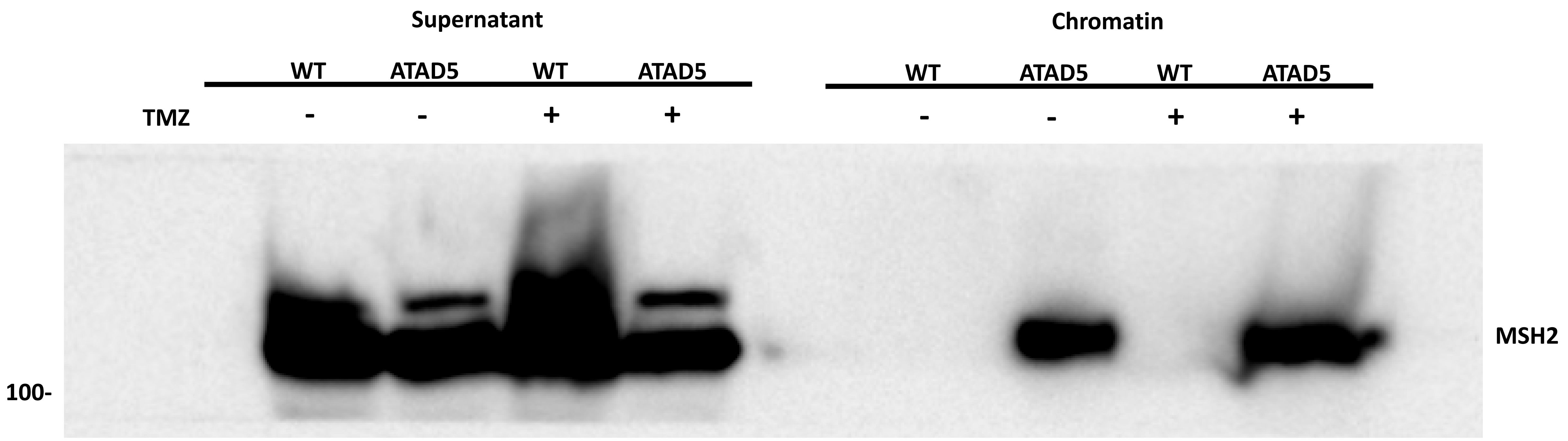

Supplementary Figure 5A

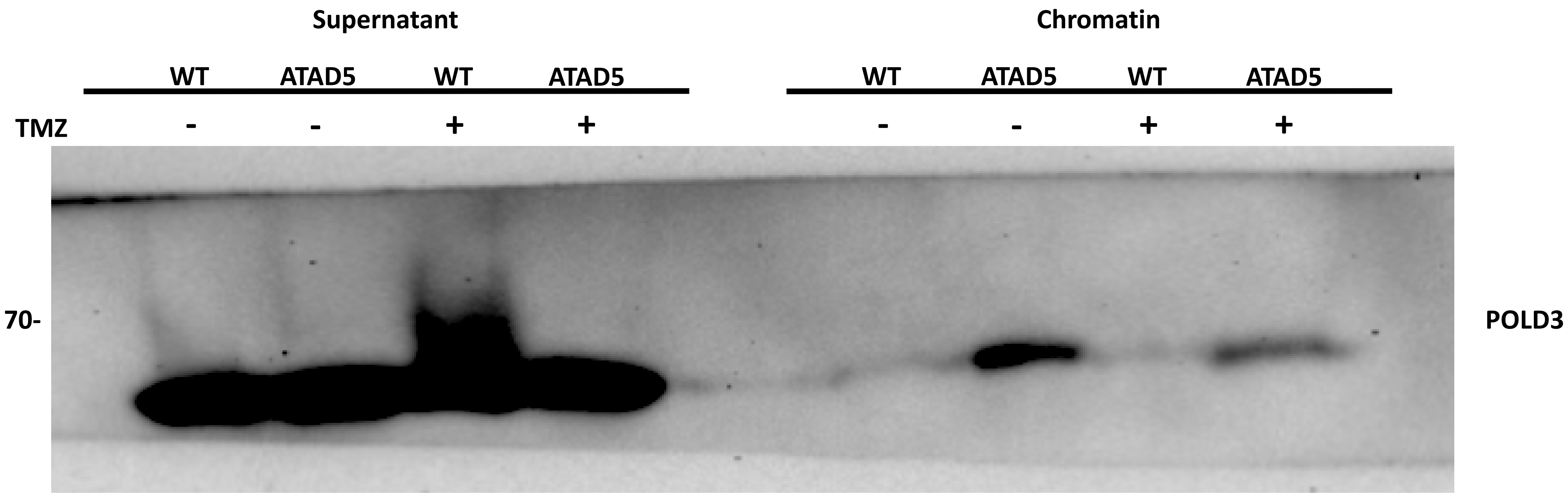

Supplementary Figure 5A

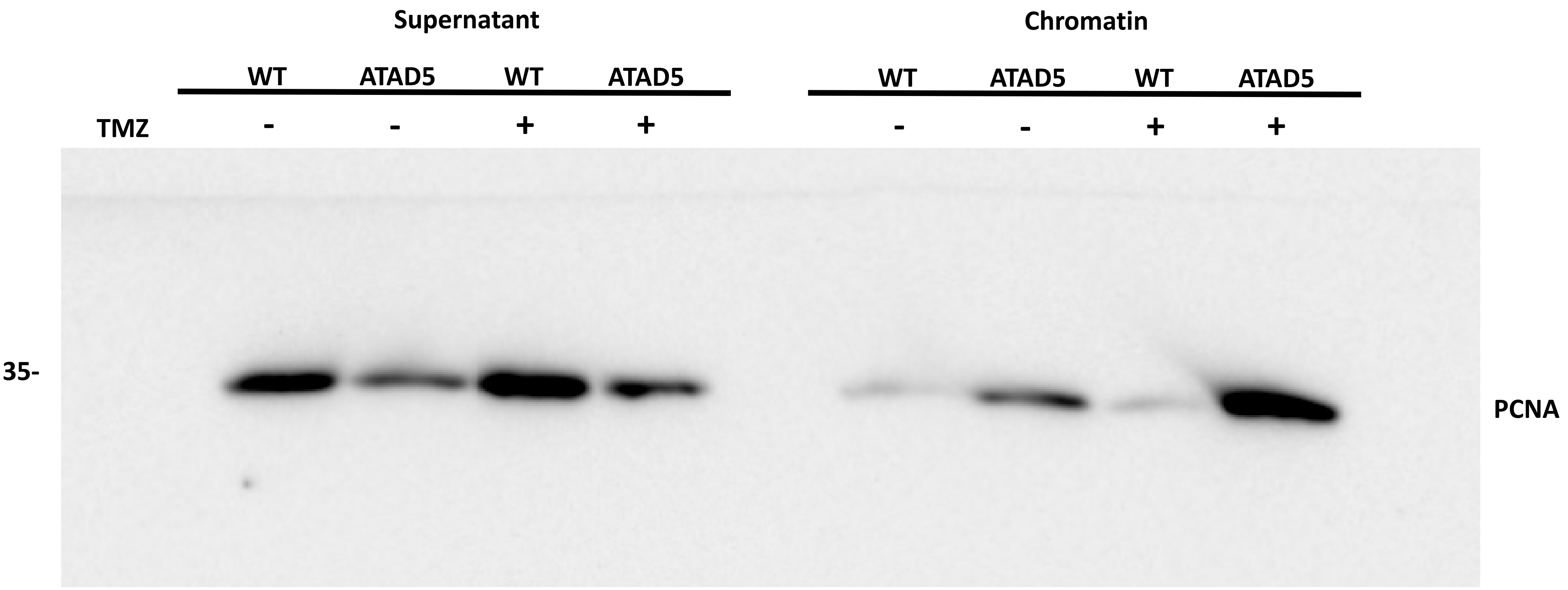

Supplementary Figure 5A

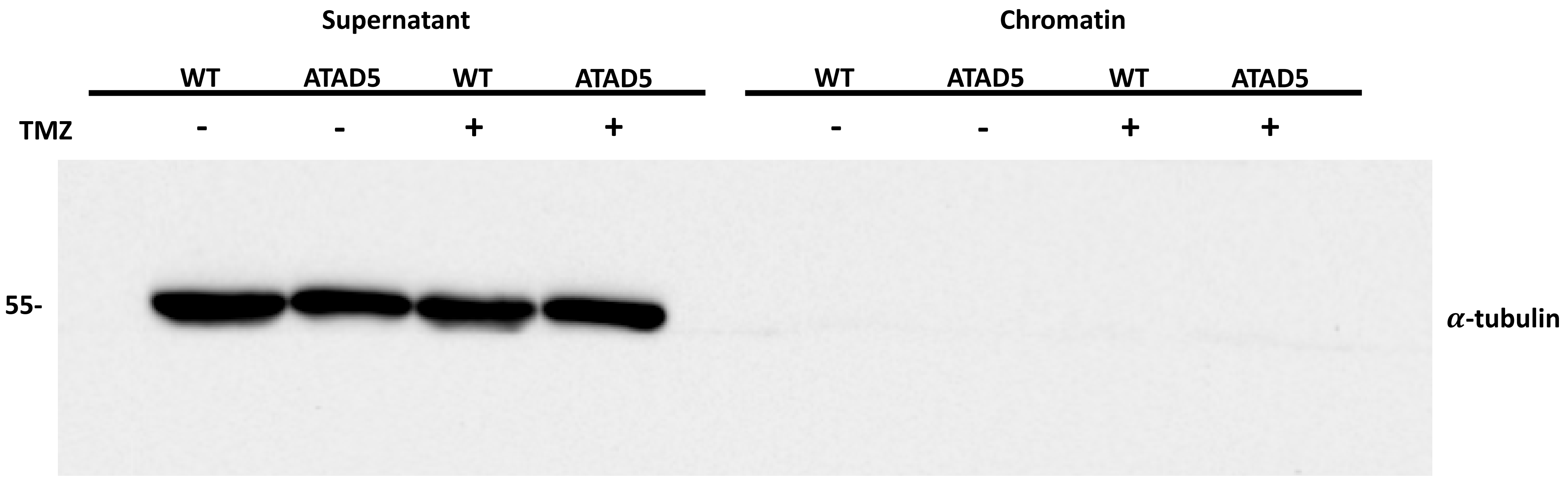

Supplementary Figure 5A

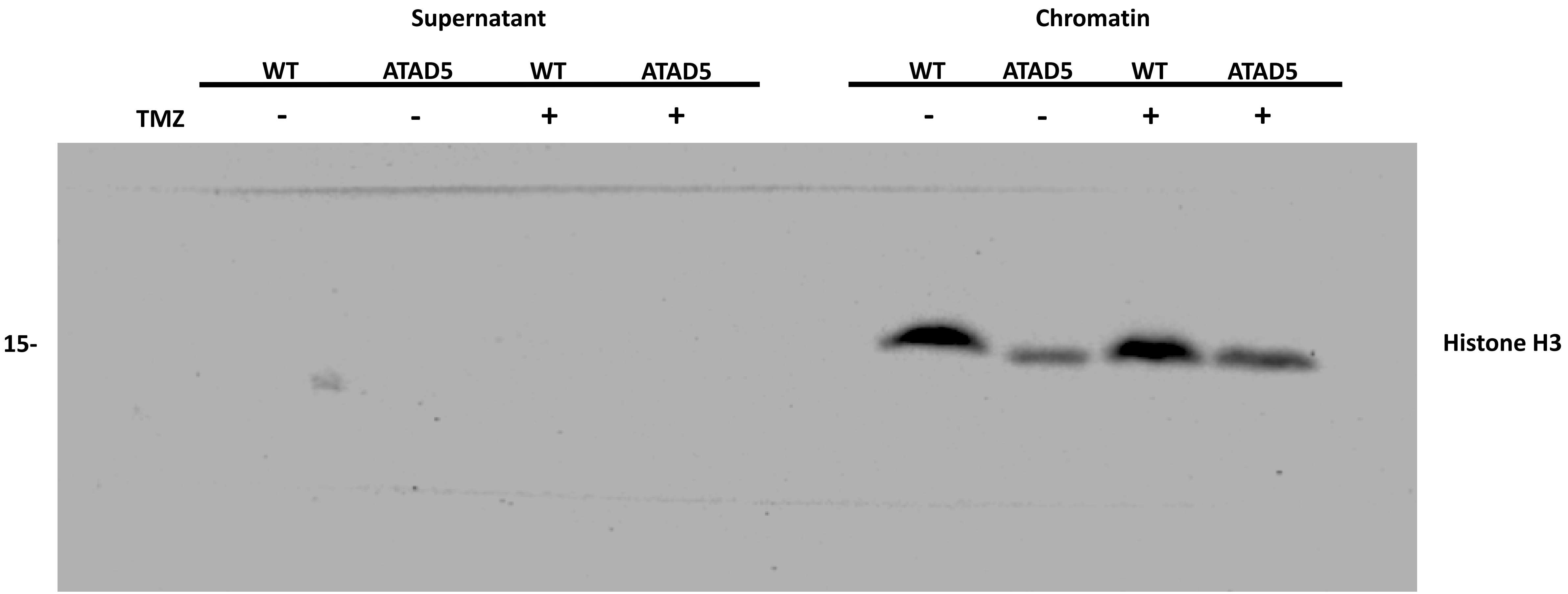

Supplementary Figure 7

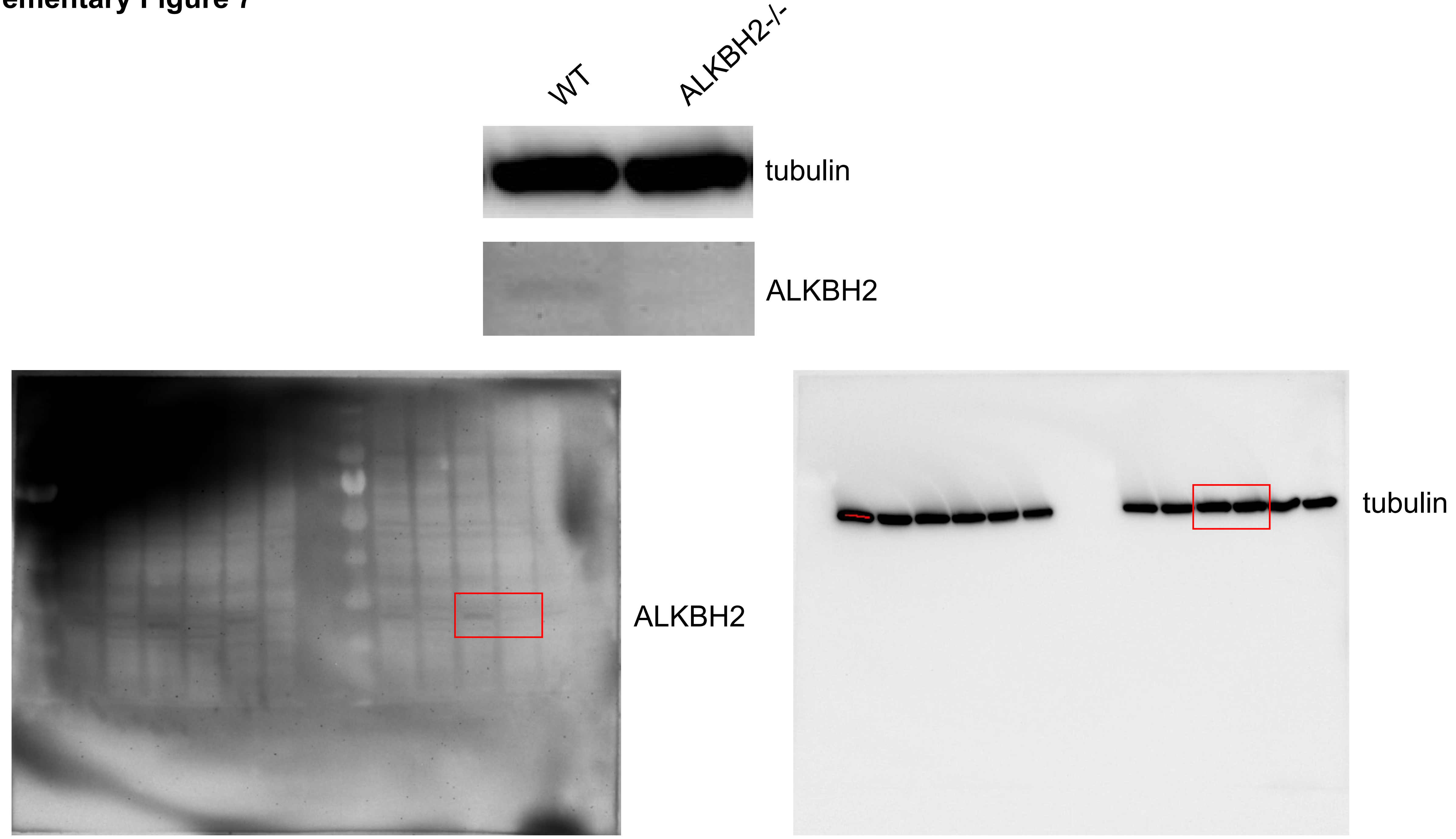

Supplementary Figure 7

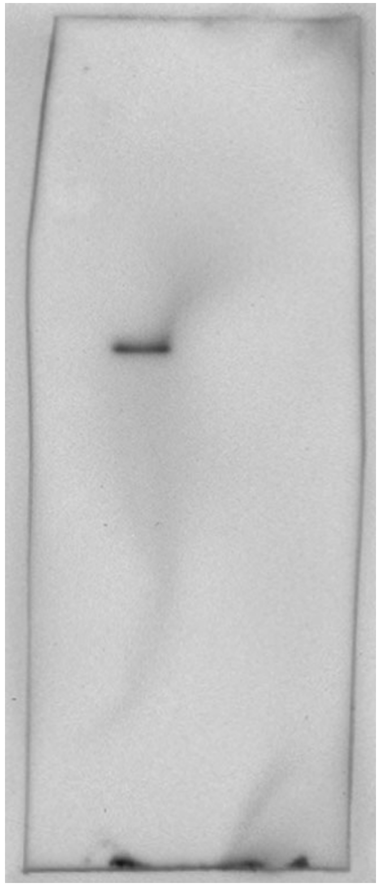

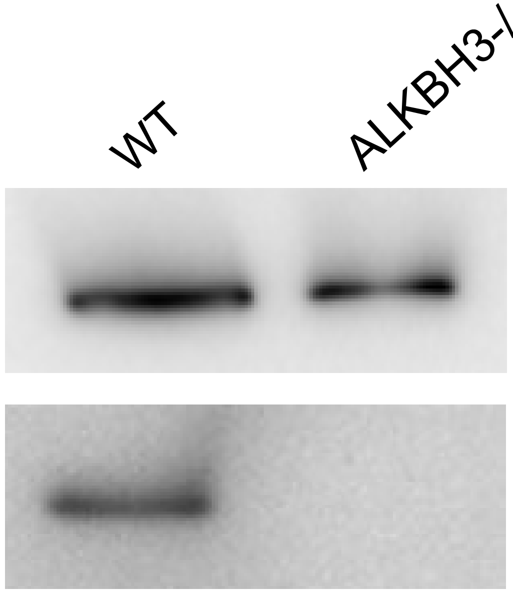

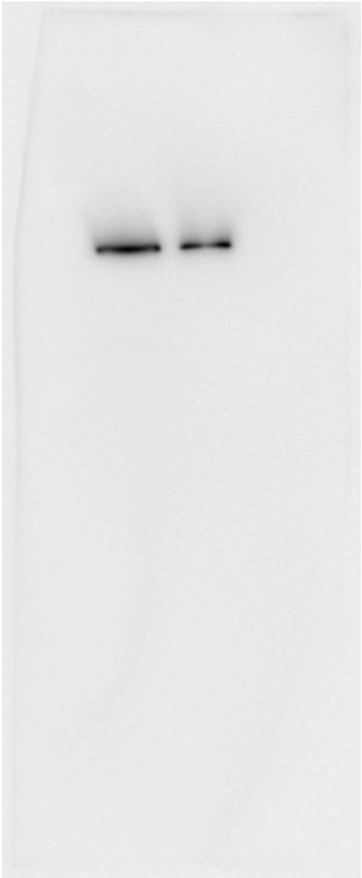

Supplementary Figure 7

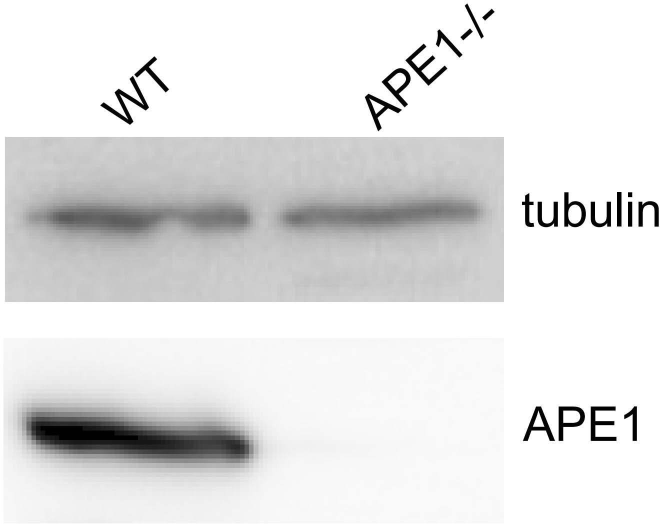

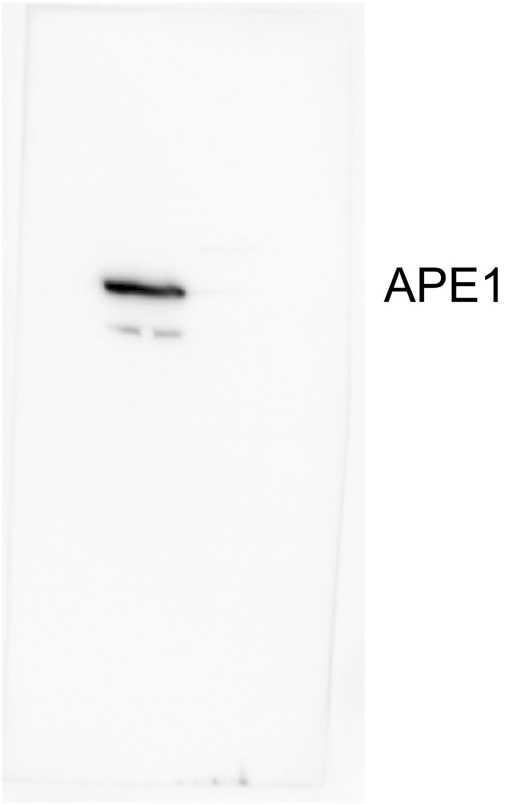

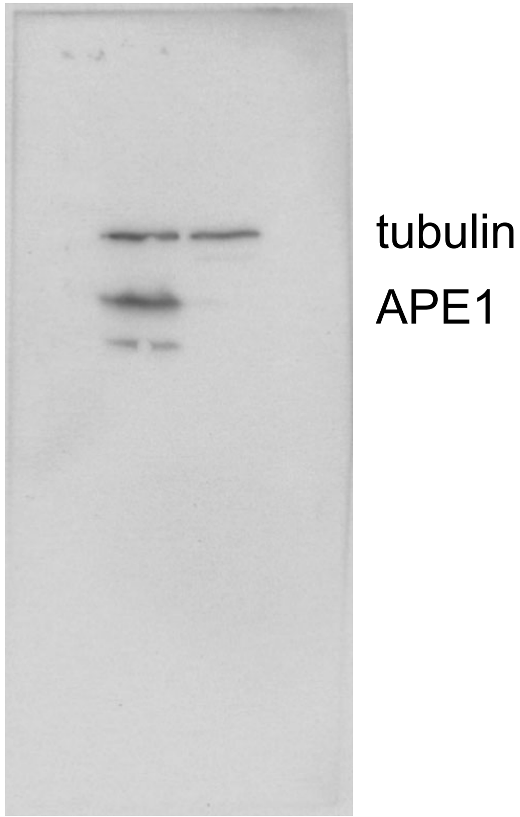

Supplementary Figure 7

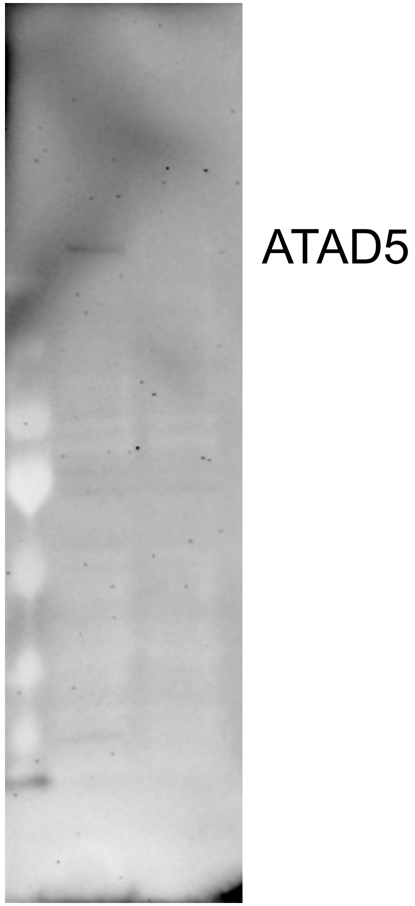

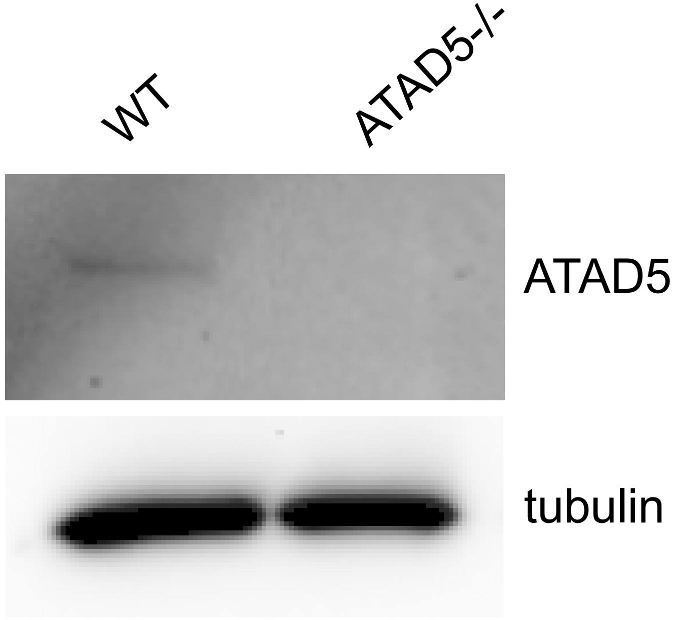

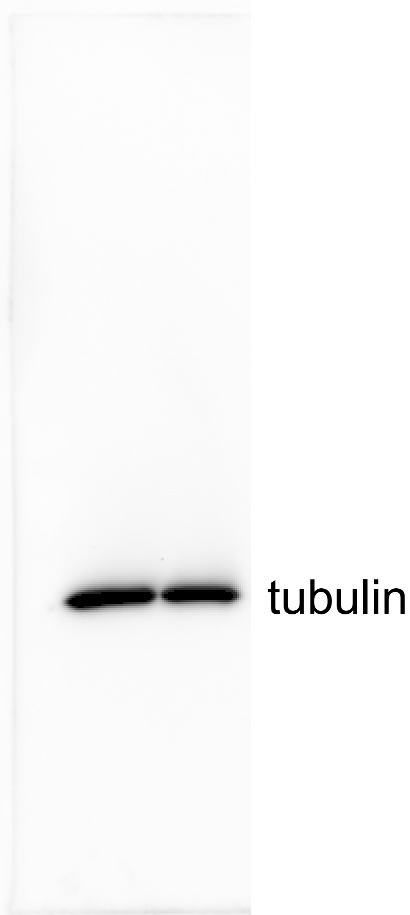

Supplementary Figure 7

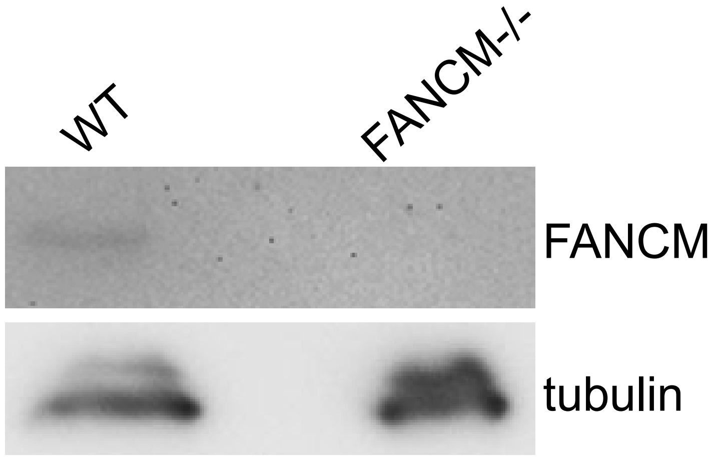

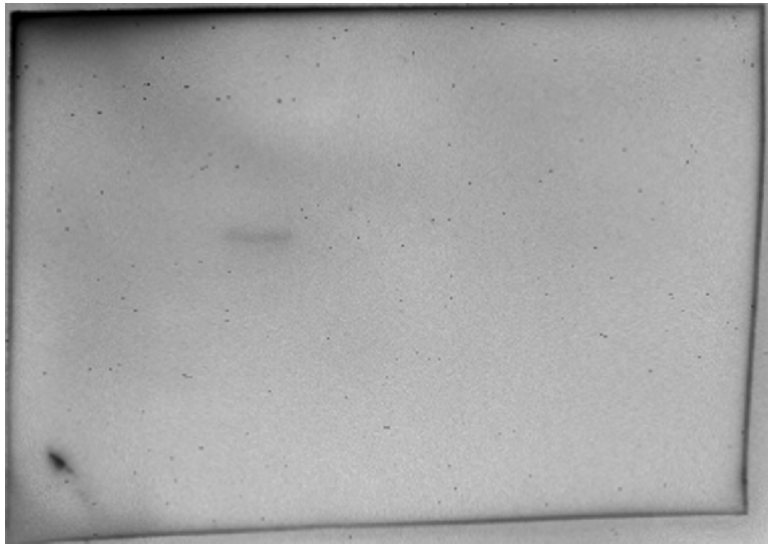

FANCM

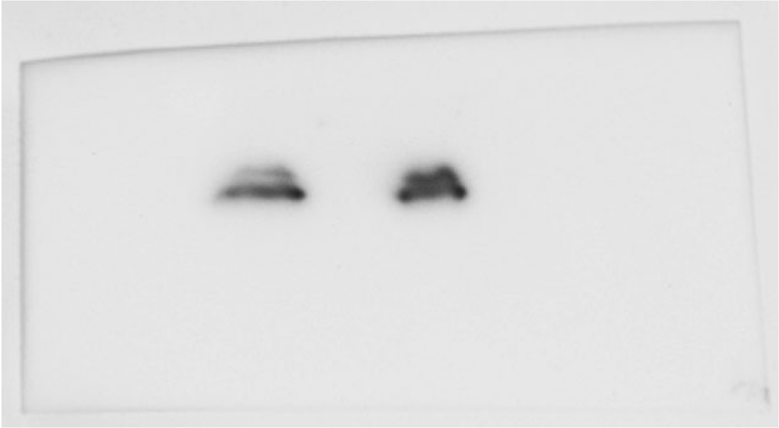

tubulin

Supplementary Figure 7

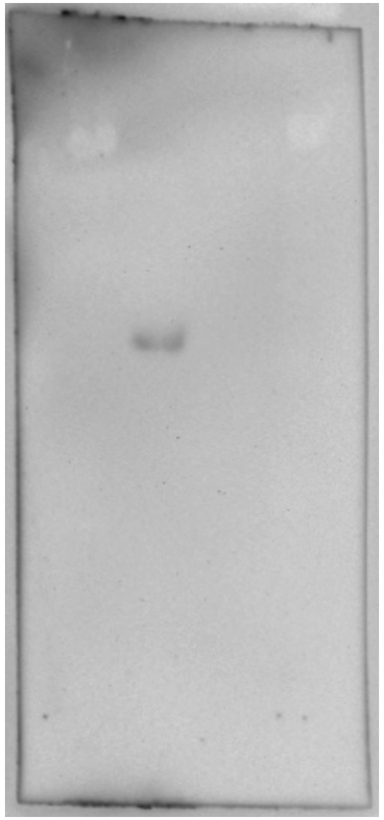

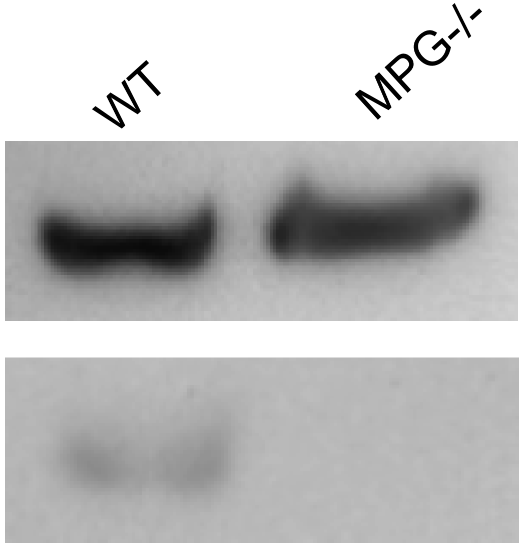

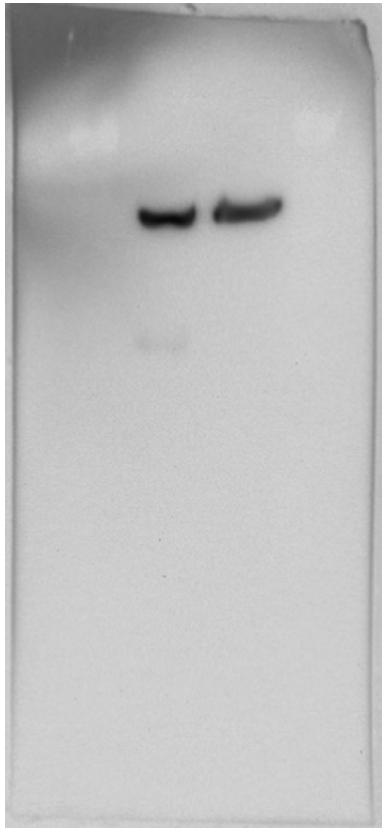

Supplementary Figure 7

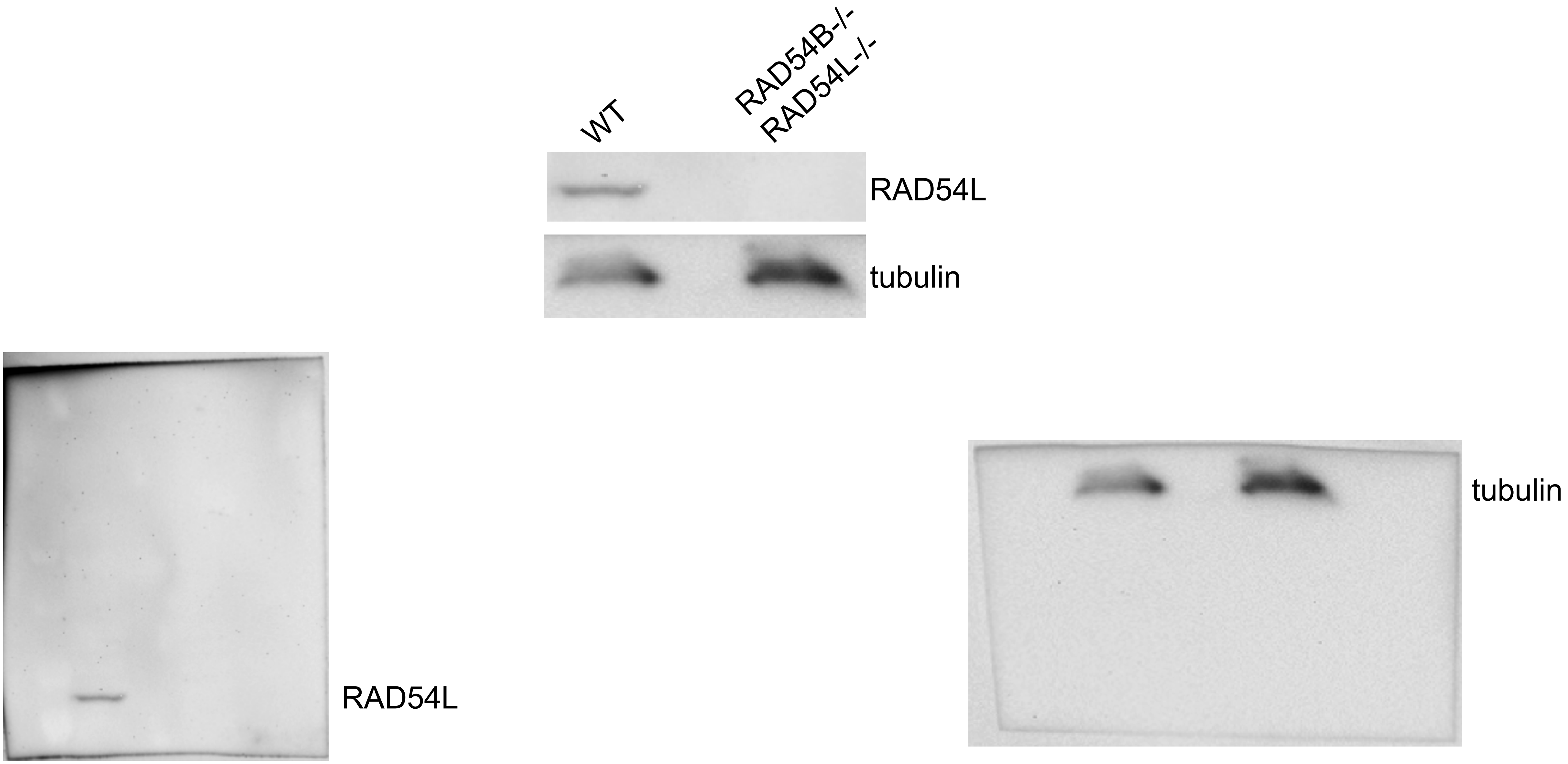

Supplementary Figure 7

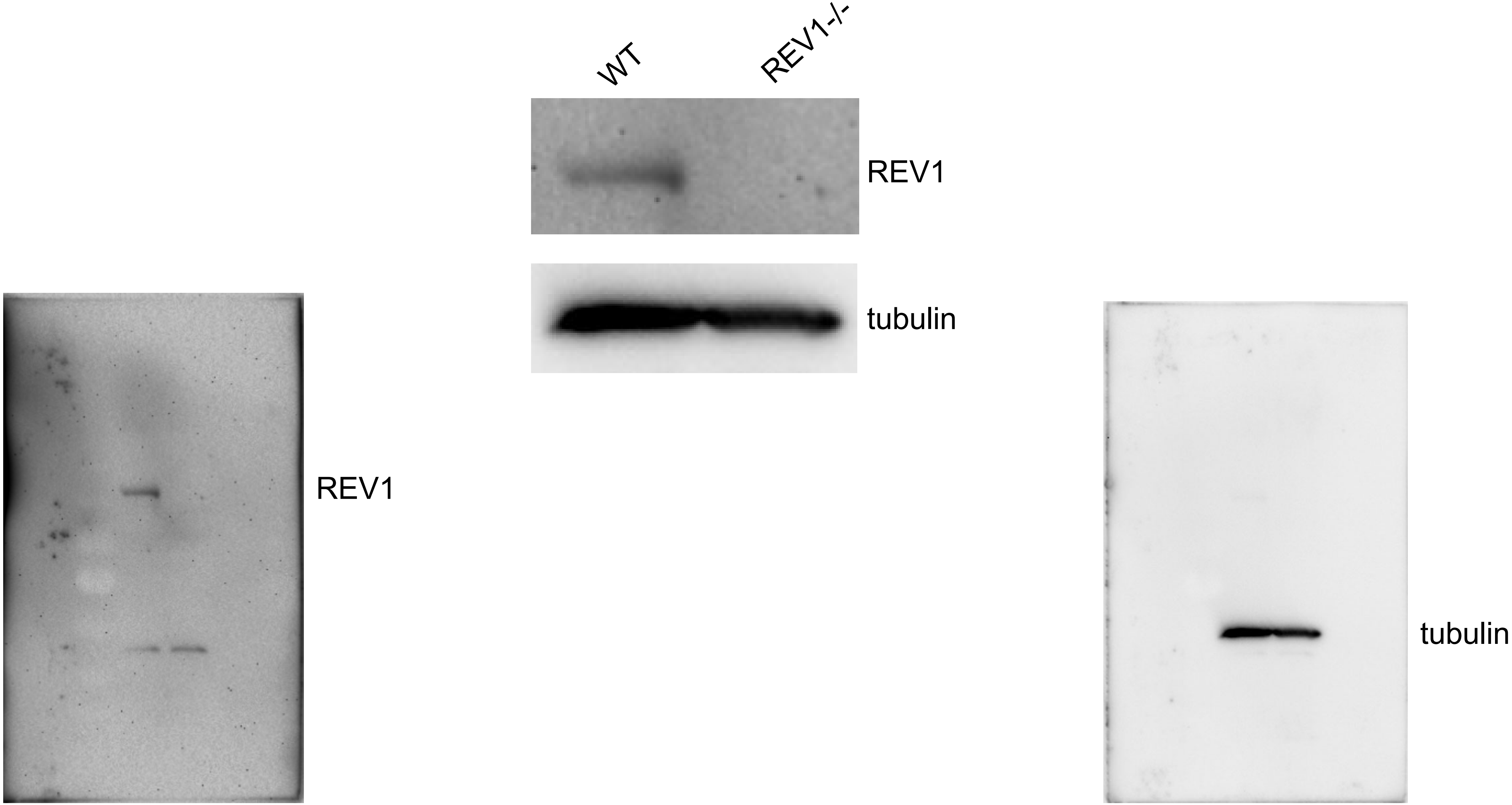

Supplement: gkae1122_Supplemental_Files [file gkae1122_supplemental_files.zip › Supplementary Material.pdf]
