# Supplementary material for: Comprehensive whole-genome sequencing reveals origins of mutational signatures associated with aging, mismatch repair deficiency and temozolomide chemotherapy
Source: Nucleic Acids Res. 2024 Dec 5;53(1):gkae1122. doi: 10.1093/nar/gkae1122 (PMC11724276; doi:10.1093/nar/gkae1122)
Supplement: gkae1122_Supplemental_Files [file gkae1122_supplemental_files.zip › Supplementary Table legends.docx]

Supplementary Table 1 Primers and antibodies used in this study.

Supplementary Table 2 Sample information and mutation counts

Supplementary Table 3 Occurrence of mutations across the entire dataset

Supplementary Material Unedited western blots
